# Regular Intake of a Usual Serving Size of Flavanol-Rich Cocoa Powder Does Not Affect Cardiometabolic Parameters in Stably Treated Patients with Type 2 Diabetes and Hypertension—A Double-Blinded, Randomized, Placebo-Controlled Trial

**DOI:** 10.3390/nu10101435

**Published:** 2018-10-05

**Authors:** Lisa Dicks, Natalie Kirch, Dorothea Gronwald, Kerstin Wernken, Benno F. Zimmermann, Hans-Peter Helfrich, Sabine Ellinger

**Affiliations:** 1 Faculty of Food, Nutrition and Hospitality Sciences, Hochschule Niederrhein, University of Applied Sciences, Rheydter Str. 277, 41065 Mönchengladbach, Germany; Lisa.Dicks@hs-niederrhein.de (L.D.); Natalie.Kirch@hs-niederrhein.de (N.K.); 2 diabetesPRAXIS Rathausallee, Rathausallee 6-8, 47239 Duisburg, Germany; doro@dr-gronwald.com (D.G.); K.Wernken@gmx.de (K.W.); 3 Department of Nutrition and Food Sciences, University of Bonn, Endenicher Allee 11-13, 53115 Bonn, Germany; benno.zimmermann@uni-bonn.de; 4 Institute of Numerical Simulation, University of Bonn, Endenicher Allee 60, 53115 Bonn, Germany; helfrich@uni-bonn.de

**Keywords:** type 2 diabetes, flavanol-rich cocoa, blood pressure, glucose metabolism, lipid status

## Abstract

Regular cocoa consumption has been shown to improve blood pressure (BP), insulin sensitivity, and lipid levels in patients with type 2 diabetes (T2D), using up to 100 g of chocolate or 54 g of cocoa. These effects, attributed to cocoa flavanols, would be beneficial for patients with T2D if they could be achieved by a usual serving size of flavanol-rich cocoa. Forty-two hypertensive patients with T2D (stable pharmacological treatment, with good adjustment for glucose metabolism, lipids, and BP) ingested capsules with 2.5 g/day of a flavanol-rich cocoa or cocoa-free capsules for 12 weeks in a double-blinded, randomized, placebo-controlled study with parallel group design. Participants had to maintain diet, lifestyle, and medication. Before and after intervention, fasting blood samples were collected; BP and nutritional status were investigated. Cocoa treatment did not affect BP, nor glucose metabolism (glucose, HbA_1c_, insulin, HOMA-IR) and lipids (triglycerides, total cholesterol, low-density lipoprotein cholesterol, high-density lipoprotein cholesterol). Body weight, fat mass, and nutrient supply remained unchanged. Changes in the placebo group did not occur. Regular intake of a usual serving size of flavanol-rich cocoa does not improve cardiometabolic parameters in stably treated patients with T2D and hypertension. As the medication modulates partly the same targets as cocoa flavanols, future studies should focus on the preventive effect of cocoa against diabetes and other cardiometabolic diseases in individuals with preexisting abnormalities that do not require any pharmacological treatment.

## 1. Introduction

Type 2 diabetes (T2D) is accompanied by an increased cardiovascular risk, partly due to comorbidities such as dyslipidemia and hypertension [1,2]. Incidence as well as the progression of these chronic diseases are strongly affected by diet. These findings explain the current interest in functional food and food ingredients which may improve cardiometabolic health [3].

Randomized controlled trials (RCTs) suggest that patients with T2D may benefit from regular cocoa consumption [4,5,6,7,8,9]. Vascular elasticity increased in patients with T2D after cocoa intake (54 g/day) [4] and in hypertensive subjects with impaired glucose tolerance (IGT) after ingestion of dark chocolate (100 g/day) [5]. In two RCTs, blood pressure (BP) was reduced after intake of 100 g [5] and 25 g [9] flavanol-rich chocolate, respectively. In another study providing cocoa (40 g/day), biomarkers of endothelial inflammation decreased [7]. Single parameters of glucose metabolism like HbA_1c_ [4,9], fasting blood glucose (FBG) [9], and insulin resistance and sensitivity [5] were improved after cocoa consumption. Favorable changes in serum lipids were also found: a decrease in low-density lipoprotein cholesterol (LDL-C) [4,5,8] and an increase in high-density lipoprotein cholesterol (HDL-C) [6,7,8] after regular intake of cocoa (20 g [8], 54 g [4]) and chocolate (45 g [6], 100 g [5]), respectively. However, these studies provided up to 100 g chocolate and 54 g cocoa daily, which cannot be recommended to patients with T2D due to the high energy content [10].

Cocoa products which provide ≥ 200 mg flavanols with a degree of polymerization (DP) of 1–10 per daily portion (2.5 g cocoa; 10 g chocolate) were approved by the European Food Safety Authority (EFSA) health claim that “cocoa flavanols (CF) help maintain the elasticity of blood vessels, which contributes to normal blood flow” [11]. Endothelial dysfunction promotes cardiovascular disorders such as atherosclerosis and hypertension in T2D [12]. Therefore, cocoa, which provides ≥ 200 mg flavanols per daily portion and little energy due to the lack of sugar and fat, may be advantageous for patients suffering from T2D.

Thus, the present study should investigate whether regular ingestion of 2.5 g/day of such an unsweetened, strongly defatted, and flavanol-rich cocoa powder might improve BP (the primary outcome measure) as well as glucose and lipid metabolism (the secondary outcome parameters) in stably treated subjects with T2D.

## 2. Materials and Methods

### 2.1. Study Design

This double-blinded, randomized, placebo-controlled trial with parallel group design was performed between September 2016 and April 2017 in a specialized medical office for diabetology (diabetes PRAXIS Rathausallee, Duisburg, Germany). The study was conducted according to the guidelines of the Declaration of Helsinki and the protocol was approved by the ethics committees of the University of Bonn (project identification code 037/16, date of approval 10 February 2016) and of the Medical Association of North Rhine (project identification code 2016143, date of approval 25 May 2016). The study was registered in the German Clinical Trials Register (DRKS-ID: DRKS00011007) on 24 August 2016. Written informed consent for inclusion was obtained from all subjects before enrollment.

Participants were consecutively recruited (August 2016–January 2017) and allocated to groups A and B (ratio 1:1) by permuted block randomization (block size of 4, sequence generated by drawing lots by an uninvolved person). They received five A- or B-capsules daily for 12 weeks. Each provided 0.5 g ACTICOA™ cocoa powder (Barry Callebaut, Zurich, Switzerland; lot no. 100-F017906-AC-796) or pure microcrystalline cellulose (J. Rettenmaier and Söhne, Rosenberg, Germany), respectively. For both treatments, nontransparent capsules of hydroxypropyl methylcellulose with identical appearance were chosen which disintegrate and dissolve quickly and completely in the upper gastrointestinal tract [13]. KPProductions (Koblenz, Germany) filled both types of capsules. The document revealing the allocation of A- and B-capsules to cocoa and placebo treatment was sealed in an envelope which was opened after statistical analysis had been finished. Thus, participants and researchers were blinded to treatment. Before and after treatment, participants were examined, and blood samples were drawn after ≥ 10 h overnight fasting. The capsules were noted in the subjects’ medication plan and were recommended to be taken in the morning (three capsules) and in the evening (two capsules) along with the prescribed long-term medication (e.g., metformin) to ensure regular ingestion. Subjects were advised not to take the capsules with milk. Furthermore, they were instructed to maintain diet and lifestyle during intervention, but to abstain from any other cocoa products and to limit the intake of further flavanol-rich foods.

### 2.2. Participants

Forty-two patients with T2D (based on the criteria of the World Health Organization (WHO) and the International Diabetes Federation (IDF) [14], diabetes duration ≥ one year) and hypertension (according to the European Society of Hypertension/the European Society of Cardiology (ESH/ESC) [15]), under dietetic and/or pharmacological treatment and with good glycemic control (HbA_1c_ 48–58 mmol/mol with consideration of individual therapeutic aims) were included in the study. Exclusion criteria comprised treatment with insulin, any changes in chronic medication in the previous three months, history of cardiovascular events, malabsorption disorders, smoking, pregnancy or lactation, present/former alcohol or drug abuse, supplementation of vitamins/antioxidants, and daily consumption of excessive amounts of other flavanol-rich foods (> one glass of red wine, one cup of green/black tea or 100 g chocolate). Eligibility was checked by questionnaire.

### 2.3. Cocoa Powder

According to manufacturer, 2.5 g ACTICOA™ cocoa provided 0.6 g protein, 0.4 g fat, and 0.6 g carbohydrates, thus delivering 38 kJ (9 kcal). The average flavanol content was 207.5 mg (8.3%) (DP 1–10). Own analysis using ultra high-performance liquid chromatography provided quantitative data on mono- and oligomeric flavanols according to their DP as well as data on individual flavanols [16] (Table 1).

### 2.4. Blood Pressure Investigation

BP was determined with a full-automatic BP monitor (OMRON Healthcare Europe, Mannheim, Germany) by a single trained investigator according to the ESH/ESC guidelines [15]. Two measurements were performed at 1- to 2-min intervals; a third measurement was done if the first two values in systolic blood pressure (SBP) differed by ≥ 5 mm Hg. The first measurement was always discarded. The second value was used for statistical analysis. If a third measurement was required, the mean value of the second and third measurement was considered.

### 2.5. Laboratory Investigations

Venous blood was collected between 7:30 and 9:00 into tubes with fluoride and citrate or tubes without anticoagulant. Fresh blood samples were analyzed by the Medical Care Center, Dr. Stein and Colleagues Laboratory Medicine, Mönchengladbach, Germany, which is accredited according to DIN EN ISO 15189. Glucose was determined in plasma. Total cholesterol (total-C), LDL-C, HDL-C, triglycerides, insulin, and creatinine were determined in serum. Glucose, lipids and creatinine were analyzed photometrically by Cobas^®^ c 701/702 and insulin by ECLIA with Cobas^®^ e 801 (both from Roche/Hitachi, Mannheim, Germany) using test kits for glucose (GLUC3; coefficient of variation (CV, < 1.3%), total-C (CHOL2; CV < 1.6%), LDL-C (LDLC3; CV < 2.0%), HDL-C (HDLC4; CV < 1.6%), triglycerides (TRIGL; CV < 2.0%), creatinine (CREJ2; CV < 2.2%) and insulin (Elecsys; CV < 2.0%). Glucose and insulin were used to calculate HOMA-IR [17]. HbA_1c_ (CV < 2.0%) was analyzed in capillary blood with the Alere Afinion^®^ AS100 Analyzer (Cologne, Germany) immediately.

### 2.6. Anthropometric Investigations

Body weight (BW), height, and waist and hip circumference as well as fat mass (FM) were investigated by a single trained examiner. Weight and height were used to calculate body mass index (BMI). Waist-to-hip ratio was determined to characterize body fat distribution according to the WHO [18]. FM was examined by bioelectric impedance analysis corresponding to the European Society for Clinical Nutrition and Metabolism (ESPEN) guidelines [19] Resistance and reactance were determined with BIA 2000-1 (Data Input, Pöcking, Germany) as described by Kirch et al. [20]. FM was computed by using the equation of Kyle et al. [21].

### 2.7. Food Intake

Before each visit, participants documented their consumption of food and beverages in standardized 3-day estimated food records (2 weekdays, 1 weekend day). The intake of energy and selected nutrients was calculated by using Prodi^®^ 6.4.0.1 (Nutri-Science, Freiburg, Germany) and the intake of epicatechin by using the United States Department of Agriculture (USDA) Database for the Flavonoid Content of Selected Foods (release 3.1 [22]).

### 2.8. Compliance

Subjects documented their capsules’ intake in a diary and returned all remaining capsules. The compliance was calculated as ratio of ingested capsules to the number which should have been ingested. On average, an intake of ≥ 4 capsules per day qualified to be included in per-protocol analysis (compliance rate ≥ 80%).

### 2.9. Sample Size Calculation

The sample size calculation was based on the estimated changes in SBP by cocoa treatment. According to a former meta-regression analysis of our group, a mean decrease of 4.3 mm Hg was expected after daily ingestion of 40 mg epicatechin with 2.5 g cocoa compared to placebo. Based on this value, we considered a mean decrease of 2 mm Hg as statistically significant [23] and clinically relevant [24]. To detect a decrease in SBP of ≥ 2 mm Hg, 16 participants per group were needed presuming a power of 75%, an alpha of 0.05, and a standard deviation of 2.8 mm Hg. The latter was calculated by weighing the variances of four studies which account for 81% of total weight in meta-analysis [25]. Assuming a dropout rate of 25%, 21 subjects were included in each group.

### 2.10. Statistical Analysis

Metric data were investigated for normal distribution by using the Kolmogorov–Smirnov test and were logarithmized if necessary. When normal distribution could be assumed, data were compared with each other by applying the *t*-test for paired and unpaired samples, respectively. Otherwise, Mann–Whitney U- or Wilcoxon test were used. Nominal data were compared by using *χ*^2^ test or Fisher’s exact test. Differences indicated by *p*-values < 0.05 were considered to be statistically significant. Metric data are presented as means and standard error of the means (SEMs) and as medians and quartiles, respectively, and nominal data are given as frequencies. Statistical analysis was performed by using IBM-SPSS Statistics, version 23.0 (IBM Corp., Armonk, NY, USA).

## 3. Results

**Figure 1:**
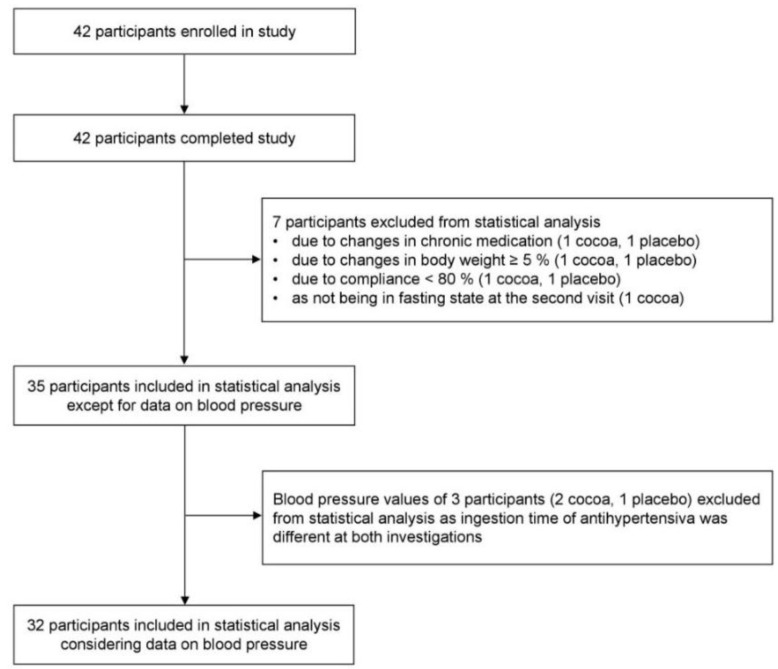
Flow of participants throughout the trial.

All 42 participants finished the study. As shown in Figure 1, one subject of each group was excluded due to changes in chronic medication (levothyroxine, cortisone) which might have affected our outcome markers, changes in BW of ≥ 5% and due to a compliance < 80%. Moreover, one participant of the cocoa group was excluded due to not being in fasted state at the second visit. Thus, 35 subjects were included in per-protocol analysis. BP values of three subjects were excluded from statistical evaluation because they had taken their antihypertensives not equally before both investigations.

The participants (18 men, 17 women) were 64.2 ± 1.5 years old and had suffered from T2D for 6.9 ± 0.8 years. Details on demographic and clinical characteristics are shown in Table 2. There were no significant differences between the groups at baseline with regard to gender, age, body height and weight, BMI, and diabetes duration.

There were no changes in BW and FM. Waist circumference (103.6 ± 4.8 cm vs. 102.3 ± 4.6 cm, *p* = 0.047) and waist-to-hip ratio (0.97 ± 0.02 vs. 0.96 ± 0.02, *p* = 0.011) decreased significantly in the cocoa group (Table 3). The intake of energy, nutrients and epicatechin from food remained unchanged throughout intervention (Table 3). The median compliance with capsules intake (%) was 99.0 (97.6; 100.0) in the cocoa group and 100.0 (98.0; 100.0) in the placebo group. Unintended effects were not reported.

As shown in Table 4, no significant differences in BP, glucose metabolism (FBG, insulin, HbA_1c_, HOMA-IR) and lipid status (total-C, LDL-C, HDL-C, triglycerides) could be detected between the groups at baseline. Changes in these parameters after both treatments did not occur (Table 4). Results on primary and secondary outcome markers of intention-to-treat analysis (*n* = 42) were not different from those of per-protocol analysis (*n* = 35).

## 4. Discussion

To the best of our knowledge, this was the first RCT which investigated the cardiometabolic effects of a usual serving size (2.5 g/day, corresponding to one tablespoon) of a flavanol-rich, unsweetened and strongly defatted cocoa for 12 weeks in hypertensive patients with T2D and stable adjustment for BP, glucose and lipid metabolism. Contrary to our hypothesis, BP, glucose, and lipid metabolism were not affected by regular consumption of 2.5 g cocoa (Table 4). Diet and nutrition status remained unchanged except for a decrease in waist circumference in the cocoa group (Table 3). However, the mean change of 1.35 cm was within the inter-measurer error of 1.56 cm [26].

Contrary to our study, two studies with a similar panel of participants showed a reduction in BP after regular ingestion of flavanol-rich cocoa products [5,9]. However, studies which found a decrease in SBP and DBP [5] or an increase in flow-mediated dilatation (FMD) [4,5] after cocoa consumption provided about 3–4 times higher amounts of epicatechin daily (203 mg [4], 111 mg [5]) compared to 40 mg in our study (not specified in [9]). In RCTs without changes in SBP and DBP, the epicatechin intake by cocoa was in a similar range (46 mg [7], 17 mg [6]) as in our study (Table 1). This can also be assumed for flavanol intake as the amount of epicatechin in cocoa products correlates strongly with the sum of catechin, epicatechin, procyanidins B2, B5, C1 and D (*R*^2^ = 0.993) [27]. Grassi et al. [28] have shown that CF dose-dependently improve BP and further vascular parameters like FMD and arterial stiffness even in amounts providing 17 mg epicatechin daily, but they investigated healthy subjects without cardiovascular risk factors and any medication. Potential confounders on BP such as changes in BW [9], body composition [5,9], or diet can be excluded in our study, but not in studies which found a reduction in BP [5,9]. Moreover, the studies of Grassi et al. [5] and Rostami et al. [9] were not double-blinded, in contrast to our study and those of Mellor et al. [6], and failed to demonstrate any effect on BP. Thus, an impact on BP due to different expectations of the participants with regard to treatment [29] can be ruled out in our study due to placebo-controlled study design.

In contrast to our study, two RCTs found an improvement in single parameters of glucose metabolism after regular cocoa consumption [5,9]. However, Grassi et al. [5] investigated subjects with IGT and observed changes in HOMA-IR and in markers of insulin sensitivity only after an oral glucose tolerance test, but not in the fasting state. In participants with T2D, changes in FBG [4,6,7], insulin [6,9], and HbA_1c_ [4,6,9] were not significant except for the decrease in FBG observed by Rostami et al. [9].

We did not find any changes in serum lipids after cocoa consumption as observed in other RCTs. However, our subjects initially had a good lipid status (triglycerides 1.7 ± 0.1 mmol/l; total-C 4.8 ± 1.1 mmol/l; LDL-C 2.9 ± 0.6 mmol/l; HDL-C 1.3 ± 0.1 mmol/l; means ± SEM) compared to those of Parsaeyan et al. [9] (triglycerides 2.6 ± 0.1 mmol/l; total-C 6.3 ± 0.2 mmol/l; LDL-C 3.5 ± 0.1 mmol/l; HDL-C 0.9 ± 0.1 mmol/l) and Grassi et al. [5] (total-C 5.5 ± 0.7 mmol/l; LDL-C 3.4 ± 0.5 mmol/l) whose status was able to be improved by cocoa. In contrast to our subjects, their participants did not receive lipid-lowering drugs [5]. Trials which did not detect changes in triglycerides nor in total-C and LDL-C investigated subjects with an adequate lipid status who were partly pharmacologically treated [6,7,9]. Results in HDL-C were different between the studies [4,5,6,7,8,9], but HDL-C is affected by lifestyle (e.g., physical exercise, changes in weight, and diet [30]) which was not sufficiently controlled in most RCTs.

Our participants were adequately treated for diabetes, hypertension, and dyslipidemia, often by using pharmacological polytherapy (Table 2). The action of some of these pharmacological agents (e.g., metformin, angiotensin-converting enzyme inhibitors, statins) is partly based on the same mechanisms that are modulated by CFs [31,32]. CFs may lower carbohydrate absorption, protect β-cell function, enhance insulin secretion, and may improve insulin sensitivity through upregulation of glucose transporters and key elements of the insulin signaling pathway [10]. Further mechanisms include lowering cholesterol absorption and synthesis, increasing nitric oxide availability, reducing endothelin-1 and inhibiting angiotensin-converting enzyme (ACE) [33].

Studies which investigated only subjects with T2D without medical treatment [8] or subjects with IGT [5,8] were able to detect a decrease in BP, HOMA-IR [5], total-C, and LDL-C [5,8]. Thus, pharmacological treatment, as simultaneously used by all of our participants, might have maximally affected the insulin signaling cascade and nitric oxide availability. This could have exhausted the potential effects of CFs. Parsaeyan et al. [8] included patients suffering from T2D and increased lipids (total-C ≥ 6.22 mmol/l; triglycerides ≥ 2.26 mmol/l), but without dietary or pharmacological treatment of hyperlipidemia. Hence, the potential for metabolic improvement by flavanol-rich cocoa products seems to be higher in individuals with some degree of dysfunction such as IGT, in insufficiently adjusted patients with T2D and in the postprandial state. Since cardiometabolic parameters did not change after cocoa treatment in pharmacologically well-treated subjects, except for an increase in HDL-C [6,7], effects by CF may occur in participants without pharmacological therapy.

BP was the primary outcome marker of our study, and daily consumption of 2.5 g of a highly flavanol-rich cocoa for 12 weeks did not affect BP in stably-treated patients suffering from T2D and hypertension. For secondary outcome markers, which did not show any effects by cocoa treatment, the study might have been underpowered.

Strengths of our RCT are the double-blinded, placebo-controlled design and the excellent compliance with treatment, probably due to encapsulation of cocoa. Adherence to dietary restrictions was controlled by investigations on nutrition status and on nutritional intake. Therefore, confounding effects by changes in lifestyle, which might have affected our outcome variables, are unlikely even if physical activity was not assessed. A limitation of our study is the lack of biomarkers for flavanol exposure such as γ-valerolactones which have shown to be the predominating metabolites of monomeric [34,35] and oligomeric [34] CFs in human plasma and in urine in the fasting state. Vascular parameters addressing endothelial function would have been interesting, but investigation was not feasible.

## 5. Conclusions

In conclusion, daily intake of 2.5 g of flavanol-rich, unsweetened and strongly defatted cocoa powder does not affect BP, glucose and lipid metabolism in stably-treated patients with T2D and hypertension in a fasting state. This may be due to pharmaceutical polytherapy which partly modulates the same molecular targets as CFs. Future studies should focus on the preventive effect of such a cocoa against diabetes and further cardiometabolic diseases in individuals with preexisting abnormalities that do not require any pharmacological treatment. This may be interesting for the fasting state as well as for the postprandial state which is associated with metabolic stress.

## Figures and Tables

**Table 1:** Nutritional and flavanol composition of the cocoa.

Ingredients	Content Per Daily Portion (2.5 g)
**Manufacturer Analysis**	
Energy (kJ/kcal)	38/9
Macronutrients	
Protein (g)	0.6
Fat (g)	0.4
Carbohydrates (g)	0.6
Micronutrients	
Sodium (mg)	0.5
Potassium (mg)	37.5
Calcium (mg)	11.4
Iron (mg)	1.1
Phosphorus (mg)	18.1
Magnesium (mg)	11.4
Methylxanthines	
Caffeine (mg)	5.0
Theobromine (mg)	52.5
Flavanols, degree of polymerization 1–10 (mg)	207.5
**Laboratory Analysis** ^a^	
Flavanols, degree of polymerization (per NP-HPLC)	
Monomers (mg)	49.7
Dimers (mg)	13.9
Trimers (mg)	5.5
Tetramers (mg)	4.7
Pentamers (mg)	3.1
Individual flavanols (per RP-HPLC)	
Epicatechin (mg)	40.4
Catechin (mg)	13.6
A-Dimers (mg)	4.3
Procyanidin B2 (mg)	12.3
Procyanidin B5 (mg)	1.3
Procyanidin C1 (mg)	3.1
Trimers ^b^ (mg)	4.1
Trimers ^b^ (mg)	4.4
Procyanidin D (mg)	4.1

^a^ Performed at the Department of Nutrition and Food Sciences, University of Bonn. ^b^ Trimeric procyanidins not further specified. NP-HPLC: normal phase HPLC; RP-HPLC: reversed phase HPLC.

**Table 2:** Demographic and clinical data.

	Cocoa group (*n* = 17)	Placebo group *(n* = 18)	*p* Baseline
Sex (*n*; %)			
Female	10 (58.8)	7 (38.9)	ns ^a^
Male	7 (41.2)	11 (61.1)	ns ^a^
Age (years)	65.6 ± 2.6	62.8 ± 1.6	ns ^c^
Diabetes duration (years)	6.7 ± 1.4	7.2 ± 1.0	ns ^c^
Antihyperglycemic drugs (*n*)			
Metformin	11	14	ns ^b^
DPP4 inhibitors	5	3	ns ^b^
SGLT2 inhibitors	2	2	ns ^b^
Antihypertensive drugs (*n*)			
Beta-receptor blockers	7	7	ns ^a^
AT1 receptor blockers	3	10	0.020 ^a^
ACE inhibitors	8	9	ns ^a^
Calcium-channel blockers	6	8	ns ^a^
Diuretics	9	10	ns ^a^
Lipid-lowering drugs (*n*)			
HMG-CoA reductase inhibitors	6	11	ns ^a^
Fibrates	1	0	ns ^b^

Data: means ± standard error of the means (SEMs) or frequencies; ^a^ *χ*^2^ test, ^b^ Fisher’s exact test, ^c^ *t*-test for unpaired samples. ACE: angiotensin-converting enzyme; AT: angiotensin; DPP4: dipeptidyl peptidase-4; HMG-CoA: 3-hydroxy-3-methylglutaryl-coenzyme A; ns: not significant; SGLT2: sodium-dependent glucose cotransporter 2.

**Table 3:** Data on nutrition status and on daily nutritional intake.

	Cocoa group (*n* = 17)			Placebo group (*n* = 18)			*p* Baseline
	Baseline	Week 12	*p*	Baseline	Week 12	*p*	
Nutrition status							
Body weight (kg)	89.9 ± 7.0	89.4 ± 7.0	ns ^c^	91.3 ± 4.6	91.3 ± 4.6	ns ^c^	ns ^a^
BMI (kg/m²) ^#^	30.2 (26.5; 34.7)	29.8 (26.3; 34.8)	ns ^c^	29.3 (26.0; 33.8)	29.5 (26.0; 33.4)	ns ^c^	ns ^a^
Waist circumference (cm)	103.6 ± 4.8	102.3 ± 4.6	0.047 ^c^	103.4 ± 2.9	103.5 ± 2.9	ns ^c^	ns ^a^
Waist-to-hip ratio	0.97 ± 0.02	0.96 ± 0.02	0.011 ^c^	0.99 ± 0.02	0.99 ± 0.02	ns ^c^	ns ^a^
Fat mass (kg)	34.7 ± 3.8	34.2 ± 3.7	ns ^c^	33.5 ± 3.0	33.5 ± 3.1	ns ^c^	ns ^a^
Fat mass (% BW)	37.7 ± 1.8	37.4 ± 1.8	ns ^c^	36.0 ± 1.9	36.0 ± 2.0	ns ^c^	ns ^a^
Nutritional intake ^§^							
Energy (kcal)	2132 ± 227	2074 ± 153	ns ^c^	1859 ± 128	2021 ± 149	ns ^c^	ns ^a^
Protein (g)	91 ± 11	85 ± 8	ns ^c^	84 ± 6	89 ± 7	ns ^c^	ns ^a^
Protein (g/kg BW)	0.8 (0.7; 1.6)	0.9 (0.7; 1.2)	ns ^d^	0.9 (0.8; 1.0)	1.1 (0.7; 1.3)	ns ^d^	ns ^b^
Fat (g)	98 ± 13	91 ± 8	ns ^c^	81 ± 6	89 ± 9	ns ^c^	ns ^a^
SFAs (g) ^#^	35 (22; 44)	36 (20; 45)	ns ^c^	31 (22; 37)	30 (25; 45)	ns ^c^	ns ^a^
MUFAs (g)	33 ± 5	29 ± 3	ns ^c^	28 ± 3	31 ± 4	ns ^c^	ns ^a^
PUFAs (g) ^#^	14 (8; 24)	15 (11; 32)	ns ^c^	14 (11; 21)	13 (9; 24)	ns ^c^	ns ^a^
Cholesterol (mg)	415 (300; 507)	401 (254; 588)	ns ^d^	389 (197; 455)	343 (254; 485)	ns ^d^	ns ^b^
Carbohydrates (g) ^#^	180 (122; 271)	198 (161; 239)	ns ^c^	169 (123; 206)	179 (143; 218)	ns ^c^	ns ^a^
Dietary fiber (g)	22 ± 2	24 ± 2	ns ^c^	20 ± 2	22 ± 2	ns ^c^	ns ^a^
Saccharose (g)	31 (22; 56)	34 (29; 52)	ns ^d^	27 (19; 43)	33 (24; 28)	ns ^d^	ns ^b^
Alcohol (g)	0.5 (0.0; 8.2)	0.6 (0.0; 4.3)	ns ^d^	0.2 (0.0; 7.7)	0.5 (0.1; 11.6)	ns ^d^	ns ^b^
Sodium (mg) ^$^	3140 ± 489	2944 ± 342	ns ^c^	3203 ± 328	3270 ± 363	ns ^c^	ns ^a^
Sodium chloride (g) ^$^	7 ± 1	7 ± 1	ns ^c^	7 ± 1	7 ± 1	ns ^c^	ns ^a^
Epicatechin (mg)	1.1 (0.3; 9.3)	4.8 (0.7; 9.4)	ns ^d^	4.9 (0.8; 6.5)	4.5 (0.8; 6.4)	ns ^d^	ns ^b^

Data: Means ± standard error of the means (SEMs) or medians (25th-percentile; 75th-percentile); ^a^ *t*-test for unpaired samples, ^b^ Mann–Whitney U test, ^c^ *t*-test for paired samples, ^d^ Wilcoxon test; ^#^ logarithmized data used for statistical tests, ^§^ based on 3-day food records performed before each investigation ^$^ salt to taste not considered. BW: body weight; MUFA: monounsaturated fatty acids; ns: not significant; PUFA: polyunsaturated fatty acids; SFA: saturated fatty acids.

**Table 4:** Data on blood pressure and on laboratory investigation.

	Cocoa group (*n* = 17)			Placebo group (*n* = 18)			*P* Baseline
	Baseline	Week 12	*p*	Baseline	Week 12	*p*	
Blood pressure							
Systolic (mmHg) ^$^	139.1 ± 3.2	138.5 ± 3.7	ns ^c^	141.6 ± 4.2	140.4 ± 4.1	ns ^c^	ns ^a^
Diastolic (mmHg) ^$^	78.1 ± 2.9	78.2 ± 2.4	ns ^c^	79.1 ± 1.8	78.2 ± 2.6	ns ^c^	ns ^a^
Glucose metabolism							
Fasting blood glucose (mmol/l)	7.6 ± 0.3	7.5 ± 0.2	ns ^c^	7.6 ± 0.3	7.8 ± 0.2	ns ^c^	ns ^a^
HbA_1c_ (mmol/mol)	46.5 (43.2; 49.7)	46.5(41.0; 50.8)	ns ^d^	47.5(44.3; 55.2)	48.6(43.2; 53.0)	ns ^d^	ns ^b^
Insulin (pmol/l)	99.6 ± 11.0	83.1 ± 9.0	ns ^c^	89.6 ± 10.1	91.8 ± 7.7	ns ^c^	ns ^a^
HOMA-IR	4.7 ± 0.5	3.8 ± 0.4	ns ^c^	4.4 ± 0.6	4.5 ± 0.4	ns ^c^	ns ^a^
Lipid status							
Total cholesterol (mmol/l)	5.0 ± 0.2	4.9 ± 0.2	ns ^c^	4.7 ± 0.2	4.6 ± 0.2	ns ^c^	ns ^a^
LDL-cholesterol (mmol/l)	3.0 ± 0.2	2.9 ± 0.2	ns ^c^	2.8 ± 0.2	2.9 ± 0.2	ns ^c^	ns ^a^
HDL-cholesterol (mmol/l) ^#^	1.3(1.2; 1.5)	1.4(1.2; 1.8)	ns ^c^	1.3(1.1; 1.4)	1.2(1.2; 1.4)	ns ^c^	ns ^a^
LDL/HDL cholesterol ratio	2.3 ± 0.2	2.1 ± 0.2	ns ^c^	2.3 ± 0.2	2.3 ± 0.2	ns ^c^	ns ^a^
Triglycerides (mmol/l) ^#^	1.3(0.9; 1.9)	1.4(0.9; 1.8)	ns ^c^	1.8(1.3; 2.3)	1.5(1.1; 2.0)	ns ^c^	ns ^a^
Creatinine (µmol/l)	61.0 ± 3.8	61.0 ± 3.8	ns ^c^	61.0 ± 3.1	61.0 ± 3.1	ns ^c^	ns ^a^

Data: Means ± standard error of the means (SEMs) or medians (25th-percentile; 75th-percentile); ^a^ *t*-test for unpaired samples, ^b^ Mann–Whitney U test, ^c^ *t*-test for paired samples, ^d^ Wilcoxon test; ^#^ logarithmized data used for statistical tests, ^$^ data refer to *n* = 15 (cocoa group) and *n* = 17 (placebo group). ns: not significant. HDL: high-density lipoprotein; LDL: low-density lipoprotein.
